# A Macroscopic Diffusion-Based Gradient Generator to Establish Concentration Gradients of Soluble Molecules Within Hydrogel Scaffolds for Cell Culture

**DOI:** 10.3389/fchem.2019.00638

**Published:** 2019-09-18

**Authors:** Anusha Dravid, Brad Raos, Zaid Aqrawe, Sam Parittotokkaporn, Simon J. O'Carroll, Darren Svirskis

**Affiliations:** ^1^Faculty of Medical and Health Sciences, School of Pharmacy, University of Auckland, Auckland, New Zealand; ^2^Department of Anatomy and Medical Imaging, Faculty of Medical and Health Sciences, University of Auckland, Auckland, New Zealand

**Keywords:** agarose, collagen, chemotaxis, neuron, biopolymer

## Abstract

Concentration gradients of soluble molecules are ubiquitous within the living body and known to govern a number of key biological processes. This has motivated the development of numerous *in vitro* gradient-generators allowing researchers to study cellular response in a precise, controlled environment. Despite this, there remains a current paucity of simplistic, convenient devices capable of generating biologically relevant concentration gradients for cell culture assays. Here, we present the design and fabrication of a compartmentalized polydimethylsiloxane diffusion-based gradient generator capable of sustaining concentration gradients of soluble molecules within thick (5 mm) and thin (2 mm) agarose and agarose-collagen co-gel matrices. The presence of collagen within the agarose-collagen co-gel increased the mechanical properties of the gel. Our model molecules sodium fluorescein (376 Da) and FITC-Dextran (10 kDa) quickly established a concentration gradient that was maintained out to 96 h, with 24 hourly replenishment of the source and sink reservoirs. FITC-Dextran (40 kDa) took longer to establish in all hydrogel setups. The steepness of gradients generated are within appropriate range to elicit response in certain cell types. The compatibility of our platform with cell culture was demonstrated using a LIVE/DEAD® assay on terminally differentiated SH-SY5Y neurons. We believe this device presents as a convenient and useful tool that can be easily adopted for study of cellular response in gradient-based assays.

## Introduction

The *in vivo* microenvironment is modulated by the precise orchestration between a multitude of chemical, physical, and biological stimuli influencing cellular behavior. Biomolecular concentration gradients established by diffusion have been implicated in a number of fundamental physiological and pathological processes occurring throughout development and adulthood i.e., embryogenesis (Christian, 2012), axonal guidance (Cao and Shoichet, 2001), and chemotaxis (Qasaimeh et al., 2018). For example, soluble concentration gradients of vascular endothelial growth factor (VEGF) have been shown to promote endothelial cell migration (Shamloo et al., 2012), interleukin-8 (IL-8) gradients are known to direct migration of human neutrophils (Vasaturo et al., 2012), whilst concentration gradients of neurotrophic factors and sonic hedgehog (Shh) have a proven role in axonal guidance (Xu et al., 2018). Yet, due to the complexity of manipulating the *in vivo* environment and associated confounding factors, the study of cellular response to gradient-based stimuli has necessitated the development of *in vitro* experimental platforms capable of generating concentration gradients in a reproducible and controllable manner.

Conventional gradient-generators such as the Boyden (Boyden, 1962), Zigmond (Zigmond, 1977) and Dunn (Zicha et al., 1997) chambers have been central in shaping our understanding of gradient-dependent cellular response *in vitro*, yet, face limitations in their inability to maintain gradient profiles beyond 1–2 h and the lack of control over gradient evolution (Keenan and Folch, 2008). Developments in microfluidic-based technology have sought to overcome the shortfalls associated with these traditional platforms by providing precise, controllable concentration gradients through manipulation of convective flow or molecular diffusion (Lin and Levchenko, 2015). However, the use of laminar flow devices associated with the former came at a cost of complexity of setup, expensive external pump systems, and potential shear stress damage to cells. Further to this, the specific expertise and intricacy required with microfluidic technology has been described as a potential hindrance for its widespread use by biologists (Sackmann et al., 2014; Lin and Levchenko, 2015). As such, there is a current dearth and necessity of simple platforms capable of generating biologically applicable concentration gradients to study cellular response.

Biopolymeric hydrogels including fibrin (Campbell et al., 2005), collagen (Vasaturo et al., 2012), and agarose (Cao and Shoichet, 2001) have previously been explored as a means to expose cells to molecular gradients formed within a three-dimensional (3D) scaffold environment in which gradient-dependent cellular response naturally occurs *in vivo*. Moreover, in both 2D “overlay” cultures and 3D cultures these hydrogels can be used to add fluidic resistance to the system, preventing mass transport by convection (Abhyankar et al., 2008). Gradients of soluble biomolecules (i.e., growth factors, cytokines) are established within the hydrogel matrix predominantly by the process of diffusion (Lühmann and Hall, 2009). Several approaches have been described in the literature: traditional methods have consisted of co-culturing the target cell or explant population alongside another cellular population transfected to release the desired biomolecule (Houchin-Ray et al., 2009). However, this method offers minimal control over gradient evolution. In another approach, the hydrogel is placed in contact with a reservoir containing the biomolecule, either as a solution or gel (Cao and Shoichet, 2001; Pujic and Goodhill, 2013). With this technique, an initial period of time is required for the gradient to form, after which a linear gradient is established across the gel. Alternative methods have involved deposition of droplets or lines of the biomolecule on the hydrogel surface either through computer-controlled micropumps (Rosoff et al., 2004) or inkjet printing (Park et al., 2017). These printing techniques, however, are limited in their inability to produce steep gradients and requirement for costly printing equipment.

In the current study, we have built upon previously described diffusion-chambers (Cao and Shoichet, 2001; Pujic and Goodhill, 2013) and present a convenient, reusable diffusion-based gradient generator capable of sustaining concentration gradients of soluble molecules within a hydrogel scaffold. Specifically, we sought to improve the stability of the gradient whilst enabling a more convenient frequency for reservoir replenishment. Two hydrogels are compared: agarose (AG) and an agarose-collagen (AG-Col) co-gel. AG is an inert, biocompatible polysaccharide comprised of alternating copolymer units of 1,4-linked 3,6-anhydro-α-l-galactopyranose and 1,3-linked β-d-galactopyranose (Jin et al., 2013; Bertula et al., 2019). The resulting hydrogel readily supports the diffusion of molecules, gases, and nutrients owing to its high porosity. However, the use of AG for cell culture is hindered by its inability to provide a bioactive extracellular matrix (ECM). Type I Collagen is a triple-helix ECM protein abundantly distributed throughout the human body. ECM proteins are widely used as scaffold materials due to their ability to interact with cells and facilitate attachment, survival, and proliferation (Ucar and Humpel, 2018). It has been shown that incorporation of Type I collagen into AG matrices can preserve the structural integrity associated with agarose, whilst providing the native ECM binding available in collagen (Ulrich et al., 2010).

Our motivations behind the present work are threefold to develop a convenient gradient generator, to establish gradients that last for days and to reduce the frequency of source-sink replenishment. Importantly, our platform confers several advantages: ease-of-fabrication without the need for lithography techniques, the ability to study both the influence of local concentration in addition to concentration gradients, and the ability to accommodate both explants and disassociated cells. We have demonstrated the capability of our device in generating diffusive concentration gradients over a period of days within both thick (5 mm) and thin (2 mm) hydrogel scaffolds. Importantly, our platform is one of the few to describe gradient formation within a co-gel matrix. We have investigated the broad applicability of our gradient-generator by studying the gradient profiles obtained with sodium fluorescein (376 Da), FITC-Dextran (10 kDa), and FITC-Dextran (40 kDa). These tracers were selected for their similarity in molecular weight (MW) to known biological molecules that interact with cells through gradient-based signaling [i.e., progesterone (314 Da) (Zhang et al., 2015), nerve growth factor (NGF, 13 kDa) (Xu et al., 2018), interleukin-8 (IL-8, 8.5 kDa) (Vasaturo et al., 2012), vascular endothelial growth factor-A (VEGF-A, 38 kDa) (Szczepkowska et al., 2012), and fibroblast growth factor-2 (FGF-2, 34 kDa) (Kole et al., 2017)]. Finally, we demonstrate the cellular biocompatibility of the device and hydrogels with SH-SY5Y neuronal cells.

## Materials and Methods

### Design and Fabrication of the Gradient-Generator

**Figure 1:**
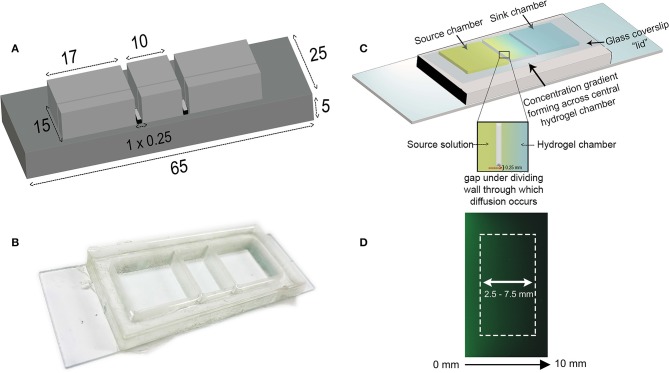
Design of the diffusion-based gradient generator **(A)** The diffusion chamber inverse master mold is designed in AutoCAD software and 3D-printed to the specified dimensions; all values are expressed as millimeters. **(B)** PDMS is cast into the mold and the chambers attached to a glass microscope slide. The device is placed in a petri dish prior to placing in the incubator. **(C)** Schematic illustration depicting the principle behind gradient formation in the diffusion-based gradient generator. A center chamber containing the hydrogel separates outer source and sink wells connected by a small 0.25 mm high gap beneath the dividing walls. The source solution diffuses through the hydrogel into the sink chamber, establishing a concentration gradient across the hydrogel. Replenishing the source and sink solutions every 24 h maintains this concentration difference between the **two** compartments. The glass coverslip “lid” is placed on top of the chambers to minimize evaporation. **(D)** Acquired fluorescent image of concentration gradient across hydrogel chamber visualized using FITC-Dextran (40 kDa) at 96 h. White rectangle illustrates the region where the gradient profiles are obtained.

A mold for the gradient-generator was designed in AutoCAD® Software and 3D printed in polylactic acid (PLA) according to the dimensions in Figure 1A. The width of the reservoirs (17 mm) was made to be broader than the central hydrogel chamber to allow for a greater volume of solution to be used than the hydrogel itself. Polydimethylsiloxane (PDMS, Sylgard® 184, Dow Corning, USA) prepared by mixing the elastomer base with the curing agent at a 10:1 w/w ratio was poured into the mold and allowed to cure at 45°C overnight. The PDMS gradient generator was detached from the PLA mold and free base polymer removed through a sequential 24 h washes in 100% hexane, 100% acetone, and 100% deionized water and followed by overnight solvent evaporation at 80°C. The gradient generator was then adhered onto a glass microscope slide (Figure 1B) using medical grade silicone adhesive (Silbione® Bluestar Silicones, NJ, USA) and left to cure overnight at 60°C. The device itself is comprised of three compartments (Figure 1C), each connected by a 0.25 mm gap, located beneath a thin dividing wall, through which diffusion of the biomolecule occurs from the source chamber to sink through the hydrogel matrix.

### Preparation of Hydrogel Solutions

A 2% stock solution of SeaPlaque^TM^ low-melting temperature agarose (melting temperature <65°C, gelling temperature 26–30°C) (Lonza, ME, USA) was prepared by dissolving powdered agarose in sterile phosphate buffered saline (PBS, Sigma Aldrich, MO, USA) in a 95°C water bath. A stock solution of 0.15% collagen was prepared on ice by diluting 3 mg/mL collagen stock solution (Type I Rat's Tail, Gibco Life Technologies) in distilled water (dH_2_O) mixed with 25 μl of 4% sodium hydroxide per mL of collagen stock and 1/10^th^ of total volume 10X PBS on ice. An AG-Col co-gel was prepared in a similar method to that described by Ulrich et al. by combining the calculated volumes of the AG and Col stock solutions made up to volume in PBS to achieve a mixture of 1% AG + 0.03% Collagen (AG-Col) (Ulrich et al., 2010). This solution was thoroughly blended by pipetting the mixture up and down, avoiding any air bubbles.

### Rheological Properties

In order to investigate whether the mechanical stiffness of the hydrogel was appropriate for neuronal culture, the hydrogels were characterized through oscillating parallel plate rheometry (40 mm diameter, 1,000 μm gap) using a Discovery Hybrid-II rheometer (USA). Briefly, the respective hydrogel solutions (1.5 mL) were prepared and loaded onto the rheometer stage (37°C). The top parallel plate was then lowered, and the hydrogel solution left to cross-link for 30 min. An initial amplitude sweep (5 rad/s) was conducted between strains 0.1–50% to define the linear viscoelastic range (LVR). Subsequent oscillatory frequency sweep experiments (0.1–100 rad/s) were conducted within this established LVR at 6% strain (AG, AG-Col) and 1% (collagen) to determine the determine the storage (*G*′), loss (*G*^′′^), and complex modulus (G^*^).

### Analysis of Intermolecular Interactions Between Hydrogels and Soluble Molecules

The interactions between AG, AG-Col, and the model molecules were investigated using Fourier-transform infrared (FTIR) spectroscopy (Bruker Optics Tensor 37, Ettlingen, Germany) in attenuated total reflectance (ATR) mode using a diamond crystal attachment. Agarose gel was prepared at 1%, matching the concentration used in the diffusion experiments. The concentration of collagen was increased to 0.1% for FTIR analysis of both pure collagen and in the co-gel to promote any signals, as the 0.03% used in diffusion experiments may not be sufficient for detection. Similarly, interactions between the soluble molecules and the gels were probed with soluble molecules at concentrations of 1 mg/ml to allow for sufficient detection. Prior to analysis, hydrogel samples were frozen at −80°C overnight and lyophilized for subsequent characterization. The spectra were obtained between the wave number range from 4,000 to 600 cm^−1^ at a resolution of 4 cm^−1^ over 32 cumulative scans.

### Characterization of the Concentration Gradients

In order to quantify the formation and stability of the concentration gradients within our device, we performed diffusion experiments using fluorescently labeled molecules sodium fluorescein (376 Da, Sigma Aldrich, MO, USA), FITC-Dextran 10 kDa (Sigma Aldrich, MO, USA), and FITC-Dextran 40 kDa (Sigma Aldrich, MO, USA) dissolved in PBS. We tested our gradient-generator across a broad range of molecular weights (376 Da−40 kDa) to validate whether our diffusion chamber would accommodate to a variety of chemoattractants. Briefly, the center chamber was loaded with either 0.3 mL (2 mm thin gel) or 0.7 mL (5 mm thick gel) of the hydrogel solution and incubated at 37°C for 40 min to allow the solution to form a gel. Prior to beginning diffusion experiments, the source and sink chambers were loaded with 1x PBS and incubated for 2 h in a 37°C cell culture incubator to ensure the gel is saturated. Without this hydration step, the gel would drain the liquid from the chambers resulting in concentration changes that were not accountable to diffusion. The PBS was subsequently removed, and the source and sink loaded with equal volumes of their respective solutions (0.5 mL for thin gel, 1 mL for thick gel). The height of the solutions in the outer reservoirs was predetermined to ensure they were not greater in height than the gel itself, minimizing the likelihood of a pressure differential forcing the source solution through the hydrogel (i.e., hydraulic gradient effect) (Offeddu et al., 2018). Every 24 h, the source and sink solutions were replenished by gently removing the old solution with a pipette, and adding new fresh solution of the same volume—ensuring that the pipette did not come into contact with the gel during this process. Fluorescence images were acquired in monochrome at 1, 24, 48, 72, and 96 h post-setup using a Leica M205 Stereomicroscope to capture the entire width of the center chamber and converted to 32-bit grayscale for analysis using ImageJ 1.50i Software (USA) (Schneider et al., 2012). The end point for this experiment was set at 96-h due to the fact that cellular response to chemical gradients is often visible from 1 to 48 h (Keenan and Folch, 2008; Atencia et al., 2012; Srinivasan et al., 2014). A 96 h time frame allowed us to extend on this period and generate a device applicable for long-term assays. The rectangle selection was used to obtain the fluorescence intensity profile within the middle 2,500–7,500 μm region of the hydrogel (Figure 1D). This region was selected to avoid interference by the meniscus and is the region where the cells would be cultured and studied. The fluorescence intensity was related to the precise concentration of molecule within the gel by means of a calibration curve, obtained by preparing different concentrations of each fluorescent molecule between 62.5 and 1,000 ng mL^−1^ mixed with the respective hydrogel solution. A second order polynomial fit was used to obtain the equation for the standard curve. Background signal of a blank hydrogel (with no fluorescent molecule) was subtracted from all images prior to analysis. As fluorescence intensity is sensitive to depth, separate calibration curves were prepared for both thick and thin gels. The absolute concentration gradient (∂c/∂x) was determined between the 2,500–7,500 μm region as the change in concentration per mm.

### SH-SY5Y Cell Line Culture and Differentiation

Undifferentiated human neuroblastoma SH-SY5Y cells were obtained from American Tissue Culture Collection (ATCC, CRL-2266) and differentiated and cultured as formerly described by Shipley et al. (2016). Briefly, the undifferentiated cells were cultured in DMEM (Gibco Life Technologies, NY, USA) supplemented with 1x penicillin/streptomycin (Gibco Life Technologies, NY, USA), 1X Glutamax (Gibco Life Technologies, NY, USA), and 15% heat-inactivated fetal bovine serum (hiFBS, Moregate Biotech, Australia) until they reached 80% confluency (~3 days). Cells were passaged once before plating for differentiation in 35 mm^2^ petri dishes. Differentiation was induced through gradual hiFBS deprivation and the introduction of Matrigel, retinoic acid (RA, Sigma Aldrich, MO, USA), brain-derived neurotrophic factor (BDNF, Peprotech, Israel) and dibutryl-cyclic-AMP (db-cAMP, Sigma Aldrich, MO, USA). Differentiated neuronal cultures were maintained in Neurobasal media (Gibco Life Technologies, NY, USA). The center compartment of the device was thinly coated with a 1:100 dilution of Matrigel in which cells were cultured to promote differentiation. Once terminal differentiation was attained (~18 days), the neurons were overlaid with hydrogel solution diluted to the appropriate concentration in Neurobasal media (Gibco Life Technologies, NY, USA). Cultivated SH-SY5Y cells were maintained below passage 10.

### Cell Biocompatibility and Morphology Study

Terminally differentiated SH-SY5Y neurons were cultured within the central chamber and overlaid with the hydrogel. Viability of the neuronal cultures overlaid with thick and thin hydrogel scaffolds was assessed after 96 h using a LIVE/DEAD® Viability/Cytotoxicity kit (Invitrogen). Additionally, the viability of cells cultured on 35 mm^2^ polystyrene petri dishes and the PDMS gradient generator (Matrigel thin-coated glass surface, no hydrogel overlay) were used as controls. Cell cultures were incubated at 37°C with 2 μM calcein-AM, 4 μM ethidium homodimer-1, and a 1:1000 dilution of Hoechst nuclear stain for 1 h. Fluorescently labeled cells were imaged using an EVOS Fluorescence Microscope (Life Technologies, 10× objective lens) equipped with DAPI (Hoechst), GFP (Calcein-AM), and Texas Red (EthD-1) filters. Two regions within the center chamber were acquired, repeated across triplicate devices (six images per condition). Individual color channels were merged using ImageJ, and the number of labeled cells counted. Percentage viability was calculated according to $\left(\frac{number\;of\;live\;cells}{total\;number\;of\;cells\;}\times 100\right)$ where the number of live cells corresponds to the cells dual labeled with calcein and Hoechst.

### Statistical Analysis

All experiments were conducted in triplicate unless specified as otherwise, and the quantitative data presented as an average ± standard deviation from the mean (SD). Statistical analyses was conducted using GraphPad Prism Software (version 8.0.2) and a two-tailed unpaired *t*-test with Welch's correction used to determine statistical significance.

## Results and Discussion

In the present work, we have described a modified PDMS diffusion chamber that can establish concentration gradients of soluble molecules within AG and AG-Col matrices. Our platform is advantageous for its ease-of-use approach to generate such gradients over a period of at least 96 h, a time frame sufficient for determination of cellular response. In this section, we present our findings and discuss the variations in the gradient profiles obtained between different fluorescent molecules (376 Da−40 kDa) and different hydrogel matrices (AG vs. AG-Col, 2 vs. 5 mm).

### Rheological Characterization

**Figure 2:**
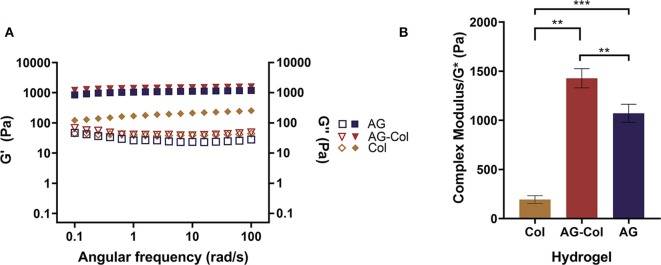
Rheological behavior of 0.2% collagen (Col), 1% agarose (AG) and 0.03% collagen+1% agarose co-gel (AG-Col) determined through parallel-plate rheometry. **(A)** Storage (shaded symbols) and loss (open symbols) moduli represent the elastic and viscous properties of the material. For all hydrogels the storage modulus exceeds the loss, indicating that the hydrogel is present in its gel state. **(B)** The complex modulus is used to represent the stiffness of the hydrogels. A statistically significant difference is observed between all groups, yet the stiffness for all remains within appropriate range for neuronal culture. Each data point represents the mean (*n* = 3) ± SD. (**p < 0.01 and ***p < 0.001).

Figure 2 depicts the rheological behavior of 0.2% collagen, 1% AG and 1%AG + 0.03%Col co-gel, respectively, as determined through parallel-plate oscillatory rheometry. It is known that hydrogels are viscoelastic materials exhibiting both liquid (viscous) and solid-like (elastic) behavior under deformation. These properties are represented by the loss (G^′′^) and storage (G′) moduli, respectively (Figure 2A). It can be seen that for all hydrogels the G′ and G^′′^ are nearly parallel trends without cross-over. Further, the G″ <G′–a typical response for cross-linked hydrogels. This suggests that the elastic properties of the hydrogel are dominant over the viscous, affirming that the hydrogels were in their cross-linked gel state throughout the rheological testing. Notably, it can be seen that for collagen, the G′ is only ~4.7× (±1.1) larger than the G^′′^, compared to AG-Col where the G′ is ~32.2 × (±5.9) greater. This suggests that even in the gel state, the mechanical strength of collagen is close to that in its viscous state. Moreover, the G′ of AG-Col is several magnitudes (~7.5×) larger than that of pure collagen—observations similar to that reported by Ulrich et al. (2010).

AG and collagen are both biopolymers that have attracted considerable interest as scaffolding materials for tissue engineering and regenerative medicine. This can be attributed to their ease of handling, non-toxicity, permeability to gases (i.e., oxygen) and small molecules (i.e., nutrients) (Tang et al., 2016). Unlike AG which is an inert, plant-derived polysaccharide, type I collagen is a native ECM hydrogel possessing bioactive ligands that interact with cellular receptors (Rosales and Anseth, 2016). At physiological conditions (37°C, pH 7) collagen monomers self-assemble to form a non-covalent entanglement of thick collagen fibrils, forming a 3D hydrogel matrix rich in topographical cues supportive to cell adhesion (Oechsle et al., 2014). Yet, despite its many advantages for cell culture, the poor physical strength of collagen remains one of its inherent drawbacks. Previously, we had conducted pilot experiments using pure 0.2% Type I Rat's tail collagen as the diffusive hydrogel scaffold within the center compartment. These trials revealed the inability of the collagen to retain its shape within the center compartment, and rather, leak into the outer reservoirs through the 0.25 mm gap beneath the dividing walls. This may be attributed to the substantially high water content of the collagen matrix and the low moduli as presented in Figures 2A,B. Therefore, we decided to utilize an AG-Col co-gel blend for the simultaneous advantages of enhancing the mechanical properties of collagen whilst providing cell attachment proteins that are lacking in AG (Bertula et al., 2019). The use of AG-Col co-gels have been formerly described to support cellular invasion, osteogenic differentiation, and neurite outgrowth (Cullen et al., 2007; Ulrich et al., 2010; Duarte Campos et al., 2015). Such composite hydrogels present as an attractive solution to overcome the paucity of biopolymeric materials offering both the necessary mechanical properties required for handling, as well as high cellular compatibility needed for long-term cell culture assays.

Cells are highly sensitive to their extracellular milieu and influenced by the mechanical properties of the hydrogel. Stiffness, a term used to refer to the intrinsic elasticity of the hydrogel matrix, has been shown to affect the migration, differentiation and regeneration of cultured cells (Hadden et al., 2017). Typically, stiffness is most pertinent for cells embedded within a 3D hydrogel scaffold. Although in our biocompatibility study the hydrogel is overlaid on top of cells adherent to a Matrigel thin-coated glass substrate, it was necessary to measure the stiffness as the gel was in direct contact with the cells. In the present study, stiffness is delineated by the magnitude of the complex modulus (G^*^) of the hydrogel (Figure 2B) Balgude et al., 2001; Cullen et al., 2007)—determined by measuring the strain (i.e., change in length) that occurs as a result of an applied stress (i.e., the force per unit area). Notably, the presence of collagen within the co-gel appears to have increased the moduli of the hydrogel (G^*^ = 1,428 ± 98 Pa) compared to AG alone (1,072 ± 91.96 Pa) (*p* = 0.0096), findings similar to that which have been reported by Cullen et al. (2007) and Kopf et al. (2016). This observation suggests that our physical blending approach of co-gel fabrication has resulted in an interpenetrated hydrogel network of the two gels likely due to hydrogen bonding between the collagen fibrils and agarose network, thereby increasing their mechanical properties. Previously, Lake et al. (2011) have shown that the addition of agarose does not alter the topology of the fibrous collagen network, whilst collagen does not hinder the gelation of the agarose gel.

In our study, we have optimized the hydrogel parameters for the culture of SH-SY5Y neurons. The brain (~1.89 kPa) (Budday et al., 2015) and spinal cord (~89 kPa) (Cheng et al., 2013) are largely compliant tissues; hence, it is expected that a hydrogel of lower stiffness is more conducive to neuronal viability. In fact, Lampe et al. (2010) have observed neuronal cells to have better survival in hydrogels with a modulus ≤3.8 kPa (Lampe et al., 2010). Both AG and AG-Col have stiffness values of <1.5 kPa, supportive of neuronal culture. Hence, we expect that our platform would be appropriate for 3D cell embedment within the hydrogel scaffold if desired.

### Analysis of Intermolecular Interactions Between Hydrogels and Soluble Molecules

**Figure 3:**
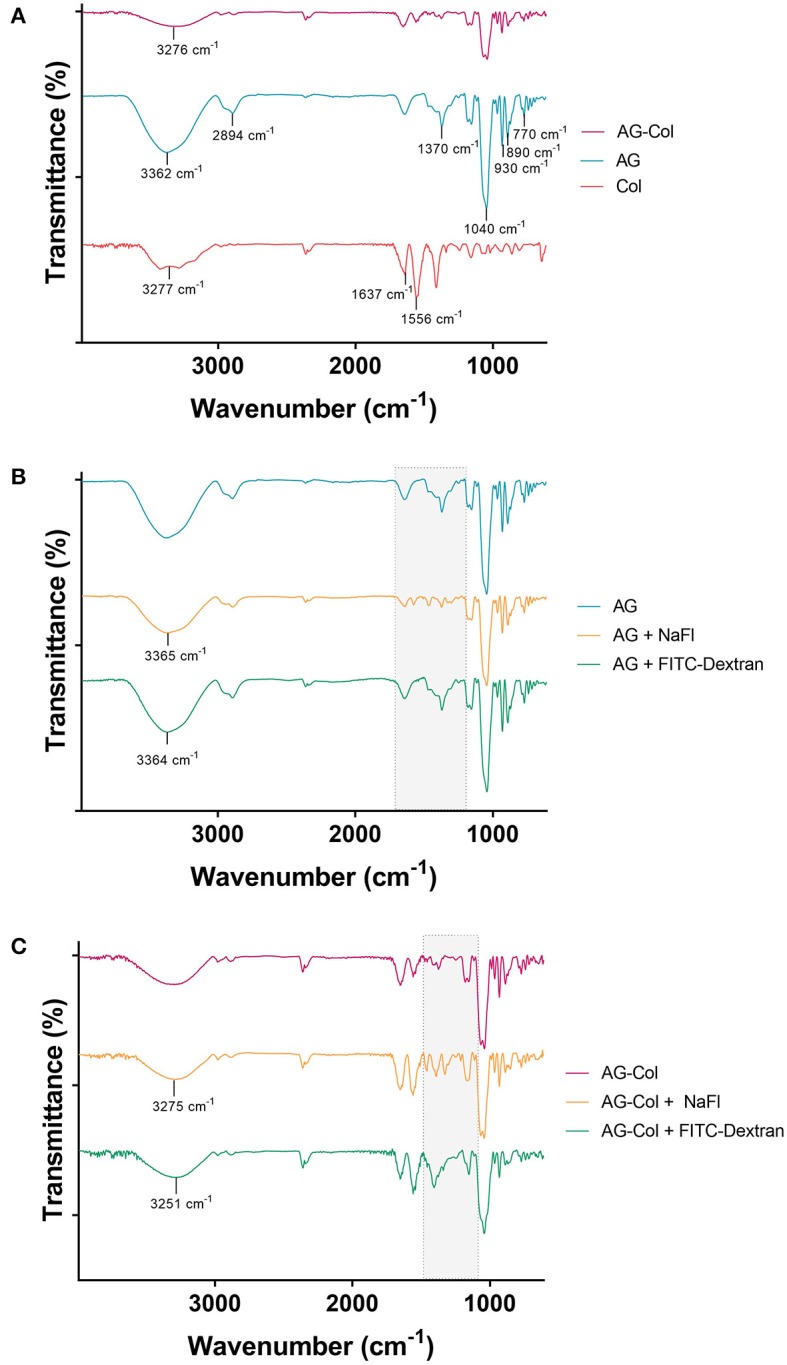
FTIR Spectra of **(A)** AG-Col, AG, Col **(B)** AG, AG + NaFl and AG + FITC-Dextran 10 kDa, and **(C)** AG-Col, AG-Col + NaFl and AG-Col + FITC-Dextran 10 kDa. Shaded “fingerprint” areas represent regions where changes and potential interactions are visible between the different spectra.

The FTIR spectra of the hydrogels and model molecules (NaFl, FITC-Dextran 10 kDa) are reported in Figure 3. The broad absorption band between 3,200 to 3400 cm^−1^ in all is ascribed to the stretching vibrations of the –OH and –NH functional groups. The presence of AG is indicated by the characteristic peaks at 3,362 cm^−1^ (-OH stretch), 1,040 cm^−1^ (glycosidic bond, C-O stretch), 2,984 cm^−1^ (-CH) and 930 cm^−1^, 890 cm^−1^ and 770 cm^−1^, which correspond to 3,6-anhydro-L-galactose skeletal banding (Tripathi and Melo, 2015). The typical bands of collagen are represented at 3,277 cm^−1^ (-NH stretch), 1,637 cm^−1^ (amide I peak, C = O stretch), and 1,556 cm^−1^ (amide II peak, -CN stretch, -NH bend). The spectrum of the composite AG-Col co-gel is signified by the combination of functional groups present in both AG (1%) and collagen (0.1%) individually (Figure 3A). However, certain peaks present in the spectra of the individual hydrogels are absent in the co-gel spectra. For example, the C-H peak at 2,894 cm^−1^ in AG is no longer present in the co-gel, suggestive of an interaction between the two polymers in the co-gel. This is further supported by the broadening of the peak at 3,276 cm^−1^; indicating some type of bonding has taken place. Figures 3B,C depict the FTIR spectra of the model molecules and either AG or AG-Col. In both, the presence of the model molecules results in some spectral changes—notably between 1,700 and 1,000 cm^−1^ (“fingerprint” region), suggesting a potential interaction between the model molecules and the gel. However, all major peaks are still evident. This is not unexpected and it should be noted that the spectra were obtained at higher concentrations of collagen (0.1%) and soluble molecules (1 mg/ml) than used in the diffusion experiments, and moreover, in a dehydrated form. In the diffusion experiments, the gels are hydrated and the soluble factors dissolved, which would be expected to reduce the significance of these interactions. Moreover, we anticipate that soluble factors (i.e., growth factors) would also interact to some extent with the hydrogels.

### Concentration Gradient Profiles in Thick (5 mm) and Thin (2 mm) Hydrogel Scaffolds

**Figure 4:**
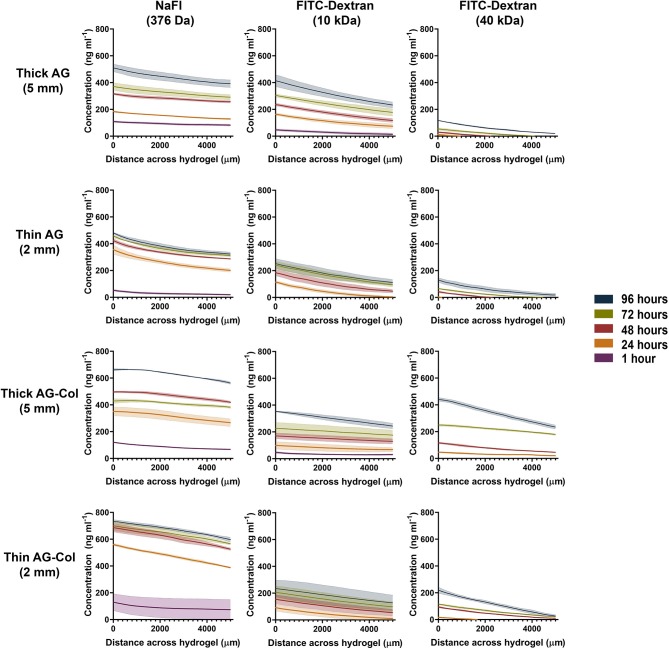
Concentration gradient profiles of NaFl (376 Da), FITC-Dextran (10 kDa) and FITC-Dextran (40 kDa) measured at various time points after setup. Concentration profile was determined across the central hydrogel chamber where 0–5,000 μm corresponds with the central 2,500–7,500 μm region of the hydrogel. The outer 2,500 μm from either end was excluded to avoid interference by the meniscus. Each data point represents the mean ± SD error bands.

The concentration gradient profiles measured at specified time-points across the inner 5 mm region of the hydrogel are presented in Figure 4. Fluorescent molecules similar in MW to known bioactive factors: NaFl (376 Da), FITC-Dextran (10 kDa), and FITC-Dextran (40 kDa) were investigated to assess whether our device could be adopted across a range of chemoattractants. By 24 h, a decrease in concentration from the source to the sink (i.e., a concentration gradient) was observed for all molecules (376 Da−40 kDa) in both thick and thin AG and AG-Col. A “pseudo steady-state” was attained by NaFl and FITC-Dextran 10 kDa by 72 h within the 2 mm thin gels; this is a phenomenon where the chemical flux entering the hydrogel equates that which is leaving (Abhyankar et al., 2006). Conversely, all molecules diffusing through the 5 mm thick hydrogels exhibited a continual increase in the absolute concentration at each time point, more evidently within the co-gel. This behavior may be due to the fact that the reservoir volumes (1 mL) were only marginally larger than the hydrogel (0.7 mL), and the height of the gel itself greater. Hence, it is possible that there was an accumulation of the fluorescent species within the gel with diffusion occurring along both the height and width of the gel (Abhyankar et al., 2006). An additional reason for this time-dependent increase in absolute concentration, may be the potential interactions between the soluble molecules and the gels as observed by FTIR (Figure 3). A similar increase in fluorescence intensity—ergo the concentration—at successive time points has been reported by Zhang et al. in their microfluidic device, used to study the chemoattractive response of spermatozoa to concentration gradients of progesterone (Zhang et al., 2015). Although the cause behind their increasing intensity is not applicable to our study, the biological observations reported still hold relevance. Despite the increase in local concentrations, the spermatozoa exhibited directed migration due to the presence of the gradient itself and the fact that the concentration range was within that which the cells are responsive to. As such, time-dependent increase in absolute concentrations does not detract from the qualitative value of the thick hydrogel for establishing chemoattractive gradients within our device as long as the absolute concentrations remain within the biological range for the factor in study.

Concentration gradients of soluble molecules are generated within our device in accordance with the source-sink model of gradient formation (Crick, 1970; Wartlick et al., 2009). These outer reservoirs contain fixed concentrations (1,000 ng mL^−1^ source, 0 ng mL^−1^ sink) in volumes larger than the central hydrogel chamber. In conjunction with periodical replenishment at 24 h intervals, it was assumed that the concentrations within these reservoirs was maintained as constant throughout the entirety of the experiment—approximating the behavior of an “infinite” source/sink. Moreover, negligible swelling (<5%) was observed in both hydrogels, suggesting that the hydrogel was sufficiently hydrated. It has also been previously reported that the fluidic resistance of the hydrogel can mitigate potential disturbance of the established gradient when the reservoirs are exchanged with fresh solutions (Abhyankar et al., 2006, 2008). Molecules have a tendency to passively move from a region of higher concentration to lower concentration. Simply put, as the gradient begins to establish, the concentration of the soluble factor will decrease as an inverse function of the distance from the source. The rate at which diffusion occurs is determined by the diffusion co-efficient (“*D*”) which in turn, is dependant on MW and the hydrogel barrier in use (Albro et al., 2009). This relationship is clearly illustrated in Figure 4 by the differences between the time it takes for the gradient to establish and the absolute concentrations attained between the three molecules. At 1 h post-setup (*t* = 0 when the source and sink chambers are loaded) a gradient of NaFl has already quickly established across the AG and AG-Col hydrogel chambers with the lowest concentration of 22.5 ng mL^−1^ present within the 2 mm AG hydrogel. In contrast, no FITC-Dextran 40 kDa could be detected at this time point. This slower diffusion rate is as expected, with FITC-Dextran 40 kDa being a molecule of MW more than 100× larger than that of NaFl. Hence, even at 24 h the concentration gradient of the 40 kDa molecule has only just begun to establish. Moreover, with increasing MW we observed lower absolute concentrations of the fluorescent molecule within the gel. For example, at 24 h (top row, thick AG) the concentration of NaFl in the gel region closest to the source was greater (~183 ng mL^−1^) than that of FITC-Dextran 10 kDa (~163 ng mL^−1^) and FITC-Dextran 40 kDa (~12 ng mL^−1^). Similar observations are seen at each successive time point, for each hydrogel presented. Interestingly, only within the thick hydrogels was a gradient of FITC-Dextran 10 kDa detectable at 1 h. In both thin AG and AG-Col the gradient begun to establish near outer edge of the gel closest to the source, outside our region of interest (data not shown). This suggests that the thickness of the hydrogel has influenced the rate of diffusion, an observation further enforced by the differing slope (i.e., steepness) of the gradients.

**Figure 5:**
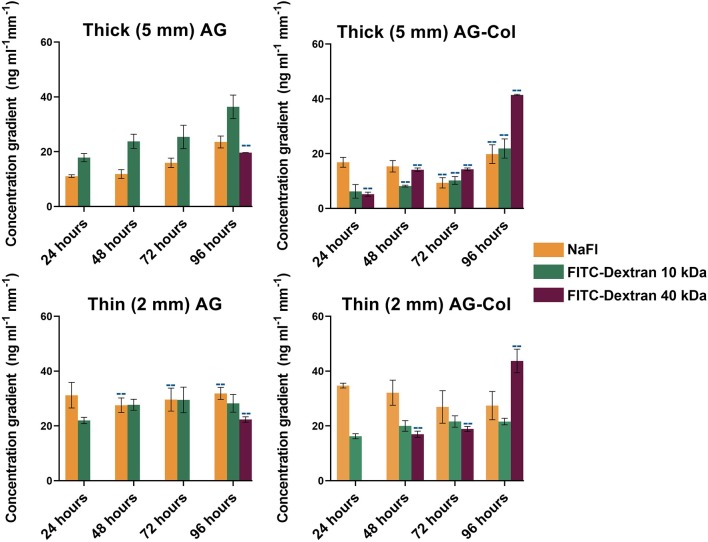
Steepness of the concentration gradient (ng mL^−1^ mm^−1^) as determined between the linear 2,500–7,500 μm region of the hydrogel chamber where cells will be cultured. Each data point represents the mean (number of replicate devices: *n* = *3*; where indicated by “**--**” *n* = 2) ± SD.

The steepness of the concentration gradients (Figure 5) was determined by measuring the change in concentration (ng mL^−1^ mm^−1^) across the 2,500–7,500 μm region, as this was the area where cells would be studied. As shown, FITC-Dextran 10 kDa displayed a notable reduction in steepness in AG-Col compared to AG alone. At 96 h in thick AG a steepness value of 36 ng ml^−1^ mm^−1^ ± 4 had reduced to 22 ng mL^−1^ mm^−1^ ± 3 in AG-Col. Similarly, the slope of the gradient had decreased by ~7 ng mL^−1^ mm^−1^ in the thin AG-Col gel compared to thin AG alone. In contrast, FITC-Dextran 40 kDa exhibited an increase in steepness between the two hydrogels (96 h, thin AG: 22 ng mL^−1^ mm^−1^ ± 1; thin AG-Col: 44 ng mL^−1^ mm^−1^ ± 4). On the other hand, the co-gel did not appear to affect the steepness of NaFl (96 h, thick AG: 24 ng mL^−1^ mm^−1^ ± 2; thick AG-Col: 20 ng mL^−1^ mm^−1^ ± 3). These observations and the lack of apparent trend suggest perhaps there may have been external perturbations that could have influenced the gradient. One such factor could be movement of the device between the incubator and the microscope for imaging which may have caused additional forces to affect the diffusive process or more significant pressure differences affecting convection through the gel. This may also account for the unexpected variations between replicates. In addition, a concentration gradient of FITC-Dextran 40 kDa in AG-Col (5 and 2 mm) was visible across the central region at 24 h, with the steepness increasing over time. This finding is in contrast to AG, where it takes up to 96 h. One potential explanation is that the presence of collagen in hydrogel has increased the pore size of the network, making it more suitable for larger molecules to diffuse. This may also provide reasoning as to why the co-gel resulted in higher absolute concentrations of NaFl and FITC-Dextran 40 kDa. The influence of scaffold thickness on the steepness of the gradient is also evident. In general, more stable gradients were observed within the thin compared to the thick gel. For example, within the thin AG the steepness of FITC-Dextran ranges between ~22 to 29 ng mL^−1^ mm^−1^, whereas for the thick AG this is between ~18 to 36 ng mL^−1^ mm^−1^. However, the steepness of FITC-Dextran 40 kDa continues to increase as the gradient gradually establishes.

There are several cell types that have been described in the literature to exhibit response to the range of steepness that can be generated by our diffusion chamber. For example, Shamloo et al. have reported a minimum NGF (13 kDa) concentration gradient of 15 ng mL^−1^ mm^−1^ to be required to direct neural progenitor cell (NPC) navigation (Shamloo et al., 2015). In a different study, it was shown that chemotaxis of human vascular endothelial cells (HUVEC) occurs in response to a minimum VEGF (20 kDa) concentration gradient threshold of 14 ng mL^−1^ mm^−1^ (Shamloo et al., 2008). For molecules ≤10 kDa, concentration gradients begin to establish within 1 h of setup. For larger molecules (40 kDa) the gradient continues to establish up till 96 h, with no gradient established in AG before 48 h.

### Cell Viability and Morphology

**Figure 6:**
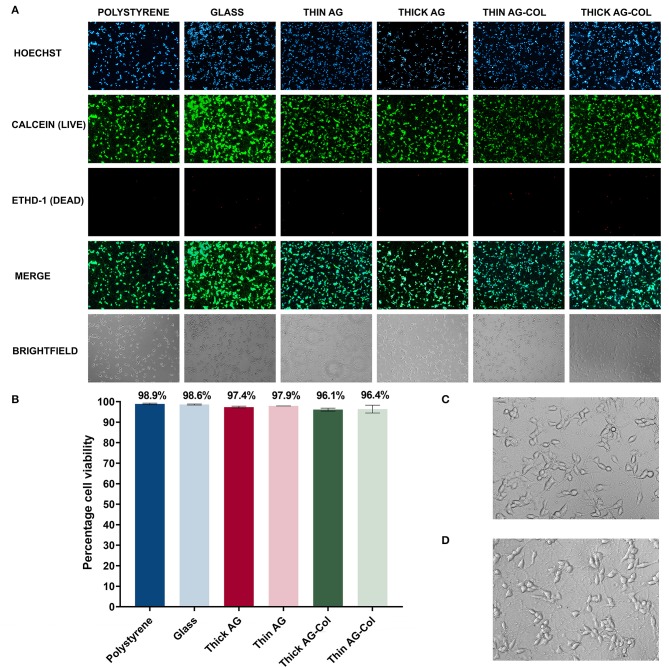
The biocompatibility of the gradient-generator and hydrogels used was investigated using a LIVE/DEAD® viability/cytotoxicity assay on SH-SY5Y neurons after 96 h in culture. **(A)** LIVE/DEAD® assay fluorescence and brightfield images visualized using a 10× objective. **(B)** The viability of the neurons was calculated by determining the number of live cells (dual labeled with Hoechst nuclear stain and calcein) as a percentage of the total number of cells. Cells grown on polystyrene and glass were used as the positive control. Each bar represents the mean (two separate regions, **three** independent replicates *n* = 6) ± SD. **(C)** Morphology of neurons cultured on Matrigel-coated polystyrene (20× objective). **(D)** Morphology of neurons cultured on the Matrigel-coated gradient-generator overlaid with thin agarose hydrogel (20× objective).

To demonstrate the compatibility of our device and hydrogels with cell culture, we investigated the viability of SH-SY5Y neuronal cells over a 96 h time frame, without a concentration gradient of chemoattractant. Phase contrast images observed with a 10× objective lens are presented in Figure 6A (last row). As shown, all cells appear healthy and project extensive neuronal processes to neighboring cells. A closer view of the cellular morphology and neurite projections as visualized through a 20× objective of neurons on the control polystyrene substrate (Figure 6C) and the gradient-generator overlaid with thin AG (Figure 6D) has been presented for comparative purposes. As observed, there was no visual difference in morphology or the extent of the neuronal projections between the two conditions. In both, the cell bodies appeared to be rounded with expansive connections to neighboring cells.

A LIVE/DEAD® viability/cytotoxicity assay was subsequently performed on the terminally differentiated SH-SY5Y neurons to assess whether our gradient-generator, inclusive of all materials and processes used, are conducive to cell survival. The viability assay was performed at the 96 h time point specifically, to correspond with the period of time cells can be exposed to concentration gradients within our device. Calcein-AM is a cell permeable esterase that labels the cytoplasm of viable cells (Figure 6A, second row) (McAdams et al., 2006) whilst EthD-1 (Figure 6A, third row) selectively labels the nuclei of dead cells (McAdams et al., 2006). Hoechst is a nuclear-specific stain labeling both live and dead cells (Figure 6A, top row). Hence, in the present study, the number of live cells was deduced by identifying those dual-labeled with both the Hoechst nuclear stain and calcein.

The biocompatibility assay revealed the ability of our platform to support cellular survival for the 96 h period. As seen in Figure 6B, the SH-SY5Y neurons maintain a high viability in all gel conditions with high viability (>96%). Cells cultured on Matrigel thin-coated polystyrene, routinely used for SH-SY5Y neuronal culture, served as a positive control for these experiments. No significant difference (*p* = 0.41 [AG]; *p* = 0.79 [AG-Col]) was observed between the 5 mm thick hydrogel (AG: 97.35% ± 0.45%; AG-Col: 96.12% ± 0.71%) and the corresponding 2 mm thin hydrogel (AG: 97.92% ± 0.02%; AG-Col: 96.37% ± 1.9%) suggesting that a 3 mm increase in hydrogel thickness did not affect cell survival. This may be attributed to the “hydrogel overlay” method we have adopted, bordering in between 2D and 3D cell culture. In this situation, fresh media and nutrients are readily accessible to the cells plated on the surface of the device, and metabolic waste products removed as the media is replenished. Moreover, the direct contact with vital ECM proteins in the thin-coated Matrigel provided the necessary attachment proteins to support the neurons throughout the 96 h culture period.

Similar observations have been reported by Cullen et al. using primary cortical neuron cultures, whereby no difference was seen in cellular viability between AG and AG-Col (Cullen et al., 2007). However, their study noted the presence of collagen within the co-gel to have enhanced neurite outgrowth compared to AG alone. O'Connor et al. have also documented greater neurite outgrowth in primary cortical neurons immobilized within collagen scaffolds compared to those in AG (O'Connor et al., 2001). Of note, the study by O'Connor et al. reports the viability of their primary cortical neurons within agarose gel scaffolds to have diminished by 35% at 24 h following plating, a finding significantly in contrast to the ~3% reduction in viability of our SH-SY5Y neurons at 96 h overlaid with AG. There are several explanations for this observation: as earlier described, during our biocompatibility studies cells were not immobilized within the hydrogel matrix, but rather the hydrogel cast on top of adherent cells plated on a thin coat of Matrigel. As such, soluble chemical molecules, including nutrients from the culture medium may reach the cells more readily and the cells were in contact with integral ECM proteins. Furthermore, we replenished both the outer reservoirs at 24 h intervals, rather than every 3–4 days allowing our cells a constant supply of fresh medium and nutrients.

## Conclusions

We have reported the fabrication and characterization of a PDMS diffusion-based gradient generator, allowing maintenance of concentration gradients within AG and AG-Col scaffolds for a period of at least 96 h. For molecules up to 10 kDa, concentration gradients begin to establish within an hour of set up, with thicker gels resulting in higher absolute concentrations. The gradient was influenced by hydrogel thickness, as the 2 mm gels resulted in steeper gradients. Both AG and AG-Col scaffolds have stiffness values <1.5 kPa, supportive of neuronal culture. The presence of collagen was seen to increase the mechanical properties of the co-gel compared to pure AG alone, suggesting an interaction between the two hydrogels in the cross-linking process which is further supported by FTIR. For the 96 h in culture, cells exhibit high viability (>96%) when overlaid with both thick (5 mm) and thin (2 mm) hydrogels. We believe this device could present as an easy-to-fabricate and convenient platform for generating long-term concentration gradients within a hydrogel scaffold for cell culture studies.

## Data Availability Statement

The raw data supporting the conclusions of this manuscript will be made available by the authors, without undue reservation, to any qualified researcher.

## Author Contributions

AD, SP, SO'C, and DS conceived the study. AD, BR, ZA, SP, SO'C, and DS had input in the experimental design. AD and BR analyzed the data. AD drafted the manuscript with support from DS and SO'C. All authors read, commented on, and approved the paper.

### Conflict of Interest

The authors declare that the research was conducted in the absence of any commercial or financial relationships that could be construed as a potential conflict of interest.
